# Spatial epidemiology of lumpy skin disease outbreaks in Thailand: proximity patterns, case concentrations, spatio-temporal clusters, and interpolated case distribution

**DOI:** 10.3389/fvets.2026.1867178

**Published:** 2026-07-23

**Authors:** Veerasak Punyapornwithaya, Supitchaya Siriyakhun, Orapun Arjkumpa, Noppawan Buamithup, Waraphon Phimpraphai, Wengui Li, Kusnul Yuli Maulana, Kenny Oriel Aranas Olana, Bolortuya Purevsuren, Karma Rinzin, Ronello Abila, Ashish Sutar, Terdsak Yano

**Affiliations:** 1 Faculty of Veterinary Medicine, Chiang Mai University, Chiang Mai, Thailand; 2 Research Center for Veterinary Biosciences and Veterinary Public Health, Chiang Mai University, Chiang Mai, Thailand; 3 The 4th Regional Livestock Office, Department of Livestock Development, Khon Kaen, Thailand; 4 Bureau of Disease Control and Veterinary Services, Department of Livestock Development, Bangkok, Thailand; 5 Department of Veterinary Public Health, Faculty of Veterinary Medicine, Kasetsart University, Bangkok, Thailand; 6 The Joint International R&D Center of Veterinary Public Health, College of Veterinary Medicine, Yunnan Agricultural University, Kunming, China; 7 Department of Veterinary Paraclinical Sciences, Faculty of Veterinary Medicine, Visayas State University, Baybay City, Philippines; 8 World Organisation for Animal Health (WOAH) Sub-Regional Representation for South East Asia, Department of Livestock Development, Bangkok, Thailand

**Keywords:** Lumpy skin disease, outbreaks, lumpy skin disease, spatial epidemiology, kernel density estimation, spatio-temporal clustering, universal kriging, Thailand

## Abstract

**Introduction:**

Lumpy skin disease (LSD) is an emerging transboundary disease affecting cattle and buffaloes, causing substantial economic and animal health consequences. Understanding its spatial dynamics is essential for formulating effective control and prevention strategies. This study aimed to characterize the spatial distribution of LSD outbreaks, identify significant outbreak clusters and interpolate the spatial distribution of LSD case values intensity across Thailand.

**Methods:**

Data on LSD outbreaks in Thailand between March 2021 and December 2021 were analyzed. Nearest neighbor analysis was employed to assess the spatial proximity among outbreak locations, while weighted kernel density estimation (KDE) was used to identify geographic areas with high concentrations of LSD cases. Additionally, space-time clusters were detected using the Density-Based Spatial Clustering of Applications with Noise (DBSCAN) algorithm combined with the Knox method. Universal kriging was performed to interpolate the spatial distribution of reported LSD case values while accounting for cattle population and spatial dependence.

**Results and Discussion:**

The results demonstrated that the mean nearest-neighbor distance between outbreak sites was 13.75 km. The KDE identified areas with high concentrations of reported LSD cases in northeastern and west-central Thailand, whereas southern and peripheral regions exhibited lower densities. The DBSCAN detected eight space–time clusters, with prominent clusters located in the northeastern, northern, and central regions. The universal kriging provided a continuous interpolation of LSD case values and revealed elevated interpolate values in the northeastern and western parts of the northern region. This study combined multiple geospatial approaches, including exploratory mapping, density estimation, spatial interpolation, and space–time cluster detection, to analyze LSD outbreak data. These spatial epidemiological insights provide explicit risk estimates and are essential for guiding efficient resource allocation, informing targeted intervention strategies, and strengthening disease control efforts in high-risk regions.

## 1 Introduction

Lumpy skin disease (LSD) is a high-impact transboundary animal disease that causes significant economic losses and is listed by the World Organisation for Animal Health (WOAH) as a priority disease requiring coordinated regional and global control efforts (1–3). It is caused by the lumpy skin disease virus (LSDV), a DNA virus belonging to the *Capripoxvirus* genus within the *Poxviridae* family (4, 5). LSD primarily affects domestic cattle and buffalo (6); however, infections have also been reported in wild ruminants, which may act as reservoirs or contribute to virus transmission (7, 8). LSD is characterized by the formation of nodules and lumps on the skin and is often accompanied by fever, edema, and lymphadenopathy. The morbidity rate of LSD varies widely, ranging from 3 to 85%, while the mortality rate is typically below 10% (6, 9). Following its first reported outbreak in Bangladesh in 2019, LSDV has spread extensively across multiple Asian countries (10–16). The economic impact of LSD outbreaks in Southeast and South Asia has been substantial, with estimated losses reaching 1.45 billion USD, largely due to livestock morbidity, production losses, and trade restrictions (17). Thailand reported its first LSD outbreaks in 2021 (18, 19).

Spatial analysis has become an essential epidemiological tool to understand and mitigate disease outbreaks by identifying spatial patterns and high-risk areas (20–22). Various analytical approaches are available for investigating disease distribution, each providing different but complementary information (23). For instance, spatial autocorrelation methods such as Moran’s *I* are commonly applied in spatial epidemiology to assess spatial autocorrelation (24–26). Meanwhile, kernel density estimation (KDE) is frequently used to identify areas of high case density, providing a smooth visualization of disease intensity (27–30). The Knox test is useful for identifying pairs of outbreak events that occur in close spatial and temporal proximity (31–34), enabling the detection of localized clustering patterns (23, 31). Furthermore, the Density-Based Spatial Clustering of Applications with Noise (DBSCAN) algorithm can refine spatial cluster identification by identifying clusters based on the density of outbreak locations, while excluding noise points (35). When integrated with the Knox test, DBSCAN enables the identification of epidemiologically connected outbreak groups based on both spatial density and space–time proximity.

In addition to identifying clusters and density patterns, spatial interpolation methods are valuable for estimating outbreak intensity at locations where outbreak observations are unavailable and generating continuous spatial prediction surfaces. Among these, kriging is a geostatistical interpolation technique that leverages spatial autocorrelation to model the underlying spatial structure and estimate values at locations where observations are unavailable (36). This approach generates a continuous spatial prediction surface, thereby enhancing the understanding of the spatial distribution and variation of disease occurrence. Previous studies have used the kriging method to explore the spatial dynamics of various infectious diseases (37–40).

Previous studies on the LSD outbreak in Thailand have largely focused on descriptive analyses (19) or applied Moran’s *I* and spatio-temporal methods (18). Most analyses were limited to the regional scale, relying on scan statistics through SaTScan (41, 42). Ultimately, exploring alternative spatial approaches can deepen the understanding of LSD dynamics. The Knox method and DBSCAN, which have not yet been applied to LSD outbreak data in Thailand, can capture overlapping or irregular clusters and detect noise and outliers, thereby providing finer insights into transmission patterns. In addition, although ecological niche modeling has been explored (43) to identify environmentally suitable areas for LSD occurrence, KDE offers a complementary perspective by characterizing the spatial distribution and intensity of outbreaks directly from case data. Kriging further extends this analysis by interpolating the spatial distribution of LSD cases values, thereby generating a continuous spatial prediction surface across the study area. Collectively, KDE, Knox, DBSCAN, and kriging provide complementary perspectives on disease occurrence by assessing spatial dependence, outbreak intensity, space–time interaction, and cluster structure. Therefore, combining multiple analytical approaches allows a more comprehensive characterization of outbreak patterns and supports more informed disease surveillance and control planning than the application of a single method alone.

To the best of our knowledge, no previous study has combined proximity analysis, kernel density mapping, cluster detection, and kriging-based interpolation of LSD case values within a single framework for investigating the spatial epidemiology of LSD in Thailand. Addressing this gap provides a more comprehensive understanding of spatial dynamics and practical insights for improving surveillance, prevention, and control strategies.

The aim of the study was to characterize the spatial proximity among outbreak events, identify areas with concentrated case occurrences, detect localized clusters that capture both spatial patterns and their temporal progression, and interpolate the spatial distribution of LSD case values across Thailand. This study offers valuable insights that support targeted surveillance, guide efficient resource allocation, and enhance disease prevention and control strategies.

## 2 Materials and methods

### 2.1 Lumpy skin disease outbreak data

Data sourced from the World Animal Health Information System (WAHIS; https://wahis.woah.org) on LSD outbreaks in Thailand were used in this study. A total of 642 outbreaks reported between March and December 2021 were included in the analysis. The dataset contained information on (i) outbreak onset dates, (ii) geographic coordinates (latitude and longitude) of outbreak locations, (iii) number of LSD cases, and (iv) cattle population at each outbreak location.

### 2.2 Data management and validation

In the WAHIS dataset used in this study, outbreak locations represented villages where outbreaks were reported. Therefore, the term “outbreak location” has been clarified as referring to village-level outbreak locations. The dataset contained information on outbreak onset date, epidemiological unit, geographic coordinates (longitude and latitude), number of cases, and population at risk.

Prior to analysis, the dataset was screened for duplicate records, missing values, and inconsistencies. Records with identical outbreak dates, coordinates, case numbers, and population values were considered true duplicates and were removed. When multiple records shared the same outbreak date and coordinates but reported different case counts, the records were aggregated into a single outbreak location to avoid duplication in the spatial analyses. No records were identified with identical coordinates associated with different outbreak dates. Furthermore, no records were identified with identical coordinates and dates but differing population values. Following data cleaning, the final dataset contained no missing values for outbreak date, geographic coordinates, number of cases, or population at risk. The geographic coordinates were examined for completeness and consistency prior to analysis. Because the coordinates represented village locations rather than administrative centroids, they were considered suitable for the national-scale spatial analyses conducted in this study.

### 2.3 Spatial distribution of LSD outbreaks

The monthly distribution of LSD outbreak locations across Thailand was analyzed and visualized using monthly maps. Additionally, outbreak locations were categorized based on the number of LSD cases and mapped using R software (44) with the “ggplot2” package to enhance spatial visualization and interpretation. The maps also incorporated publicly available elevation data from the NASA SRTM Digital Elevation Model to depict the terrain features of the study area (45).

### 2.4 Spatial proximity based on nearest neighbor distance

To determine the spatial proximity characteristics of LSD outbreak point data, the pairwise closest distances between each outbreak location and its nearest neighboring outbreak were calculated using the *distHaversine* function from the “geosphere” package in R (46).

For each outbreak location i , the nearest neighbor distance was determined by excluding the self-distance (set to infinity for i=j ) and identifying the minimum distance to any other location j . This was achieved by iterating all locations and calculating pairwise distances. Subsequently, the average nearest neighbor distance (AVND) was computed using Equation 1 (46).


$$\text{AVND}=\sum_{i=1}^{n}\min_{j\neq i}(\text{dist}(i,j))$$ (1)

where, n is the total number of outbreak locations and dist(i,j) represents the distance between points i and j . The $\min_{j\neq i}$ function identifies the nearest neighbor to point i , and the average nearest neighbor distance is obtained by dividing the total by n .

This method provides an estimate of the spatial clustering of LSD outbreaks and is useful for understanding the proximity between outbreak locations within the study area. Notably, a previous study using a similar WOAH dataset identified positive spatial autocorrelation through Moran’s *I* and Moran’*I* indices within the local indicators of spatial association (LISA) (18). Therefore, the present study did not aim to replicate the analysis but rather to reference its findings.

### 2.5 Spatial density based on KDE

A weighted KDE was performed using all outbreak locations and corresponding case counts to identify areas with high concentration of LSD cases across Thailand. This method assumes that values closer in space tend to be more similar. Consequently, the spatial correlation observed among outbreak locations provides the basis for interpolating reported LSD case values throughout the study area (40).

In weighted KDE, the density $\hat{f}(s)$ at a given location s was estimated using Equation 2.


$$\hat{f}(s)=\frac{1}{nh^{2}}\sum_{i=1}^{n}w_{i}\cdot K(\frac{|s-s_{i}|}{h})$$ (2)

In this equation, n is the total number of observed points, and $s_{i}$ denotes the spatial coordinates of the $i^{\mathrm{th}}$ point, and $w_{i}$ refers to the number of LSD cases reported at location $s_{i}$ , serving as the weight in the calculation. The parameter h is the bandwidth, which determines the level of spatial smoothing.

The effectiveness of KDE is highly sensitive to the choice of bandwidth (*σ*) because this parameter determines the degree of spatial smoothing applied to the data. In this study, outbreak points were weighted by the number of cases, and bandwidth selection was guided by a data-driven approach using the *bw.diggle* function from the “spatstat” package (47). This method is specifically designed for inhomogeneous or weighted point patterns, identifying the bandwidth that best reflects the underlying intensity structure. By optimizing the bandwidth using this technique, the analysis minimizes subjective bias and enhances the reliability of spatial risk estimation.

The KDE surface representing the concentration of LSD cased was generated using a combination of the “ggplot2” and “sf” (48). The shapefile for Thailand’s country boundaries was obtained from the Open Development Thailand (ODT) public repository (49).

### 2.6 Space–time clusters of LSD outbreaks

In this study, spatial and temporal thresholds for clustering were defined using the Knox method and previous study (23, 50, 51). A spatial threshold of 50 km was applied based on the outbreak-focused zone specified by the Thai livestock authority (52). A temporal threshold of 30 days was chosen to align with the LSD incubation period (28 days), as defined in the WOAH Terrestrial Animal Health Code (Chapter 11.9, Article 11.9.1) (53) and Thailand’s livestock surveillance and monthly reporting practices (54).

Spatial and temporal proximity between outbreak events was assessed using Knox-type threshold criteria. First, the outbreak date was converted to the number of days from the earliest reported outbreak date. Pairwise temporal distances were then calculated in days, while pairwise spatial distances between outbreak locations were calculated in kilometers using the Haversine formula. A spatial threshold of 50 km and a temporal threshold of 30 days were applied.

To combine space and time within a single metric, both distance matrices were standardized by their respective thresholds. Specifically, spatial distance was divided by 50 km, and temporal distance was divided by 30 days. In this study, the Knox framework was used to define epidemiologically meaningful spatial and temporal proximity thresholds and to construct the standardized space–time distance matrix. A formal Knox test of statistical significance was not performed. Instead, the standardized distance matrix was used as the input for DBSCAN clustering.

Following construction of the standardized space–time distance matrix, clustering was performed using the DBSCAN algorithm implemented in the dbscan R package (55). The neighborhood radius parameter (*ε*) was determined from the distribution of the combined standardized space–time distances. Specifically, *ε* was set to twice the median value of the combined distance vector, with a minimum value of 0.1 to prevent excessively small neighborhood sizes. This approach allowed outbreak locations that were sufficiently close in both space and time to be assigned to the same cluster while preserving the ability to detect localized outbreak groups.

The minimum number of neighboring outbreak locations required to form a cluster (minPts) was set to 2. This threshold was selected to enable detection of small but epidemiologically relevant outbreak clusters while allowing isolated outbreak locations that did not satisfy the density requirement to be classified as noise. The DBSCAN algorithm was then applied to the standardized space–time distance matrix to identify density-connected outbreak clusters.

DBSCAN identified clusters by grouping outbreak events that were close in the combined space–time metric, while outbreak events that did not meet the density-based clustering criteria were classified as noise. The significance of DBSCAN clusters was not evaluated using *p*-values, because DBSCAN is an unsupervised density-based clustering algorithm rather than a hypothesis-testing method. Instead, the robustness of the clustering results was evaluated through sensitivity analysis.

To evaluate the robustness of cluster identification to temporal threshold selection, additional analyses were performed using temporal thresholds of 15 and 45 days while maintaining the same spatial threshold (50 km) and DBSCAN parameter settings as used in the primary analysis. This approach enabled assessment of the influence of temporal threshold selection on cluster membership and cluster structure while minimizing the confounding effects of changes in clustering parameters.

### 2.7 Spatial interpolation of LSD case values using universal kriging with a population covariate

Universal kriging was applied to predict the spatial distribution of LSD case values while accounting for variation in cattle population through the inclusion of a population covariate. This geostatistical method models the observed value at each location Z(s) , as the sum of a deterministic trend μ(s) , and a spatially autocorrelated residual ε(s) , as expressed in Equation 3 (56):


$$\begin{array}{c} Z(s)=\mu (s)+\varepsilon (s) \end{array}$$ (3)

The deterministic component μ(s) captures broad-scale variation and is expressed as a linear function of covariates (Equation 4) hypothesized to influence disease risk (57).


$$\begin{array}{c} \mu (s)=\beta_{0}+\beta_{1}\cdot x(s) \end{array}$$ (4)

In this study, the covariate x(s) represents the animal population at each location. Incorporating cattle population into the trend component allows universal kriging to account for spatial heterogeneity associated with livestock distribution, thereby improving the prediction of case values and generating a more representative interpolated case surface. To model spatial dependence, the semivariogram was quantified and fitted using Equation 5 (40, 58).


$$\begin{array}{c} \hat{\gamma }(h)=\frac{1}{2n(h)}\sum_{i=1}^{n(h)}\begin{array}{c} {\left[z(x_{i}+h)-z(x_{i})\right]}^{2}\\ \; \end{array} \end{array}$$ (5)

where $\hat{\gamma }(h)$ denotes the experimental semivariogram, h is the lag, $z(x_{i})$ represents a measurement taken at location $x_{i}$ , and n(h) refers to the number of pairs of h units apart in the direction of the vector.

The semivariogram was analyzed to quantify spatial autocorrelation among LSD outbreak locations, which in turn guided the selection of an appropriate theoretical model for universal kriging. An empirical semivariogram was first constructed based on the LSD outbreak data to evaluate the spatial dependence of observations at varying distances. Three theoretical models, including spherical, exponential, and Gaussian–were tested (59, 60). These models were evaluated using a grid search across combinations of variogram parameters, including nugget, sill, and range, and assessed using leave-one-out cross-validation (60). Model performance was measured using two criteria: Root Mean Square Prediction Error (RMS), which assesses the difference between predicted and observed values, and Average Standard Error (ASE), which evaluates internal prediction consistency (61). The optimal variogram model was selected based on the lowest RMS and the smallest absolute difference between RMS and ASE. This process ensured that the chosen model accurately represented the spatial structure of the data and was suitable for universal kriging interpolation.

Functions from the “*gstat*” package (62) were used to calculate the empirical semivariogram and fit a theoretical variogram model using a grid search approach. Furthermore, the *krige* function was used to perform universal kriging, whereas the *krige.*cv. function was used to conduct LOOCV. Additionally, the results were visualized using the “*ggplot2*” and “*sf*” packages (48), generating maps with color gradients that depict the spatial distribution of interpolated LSD case values across Thailand.

**Figure 1:**
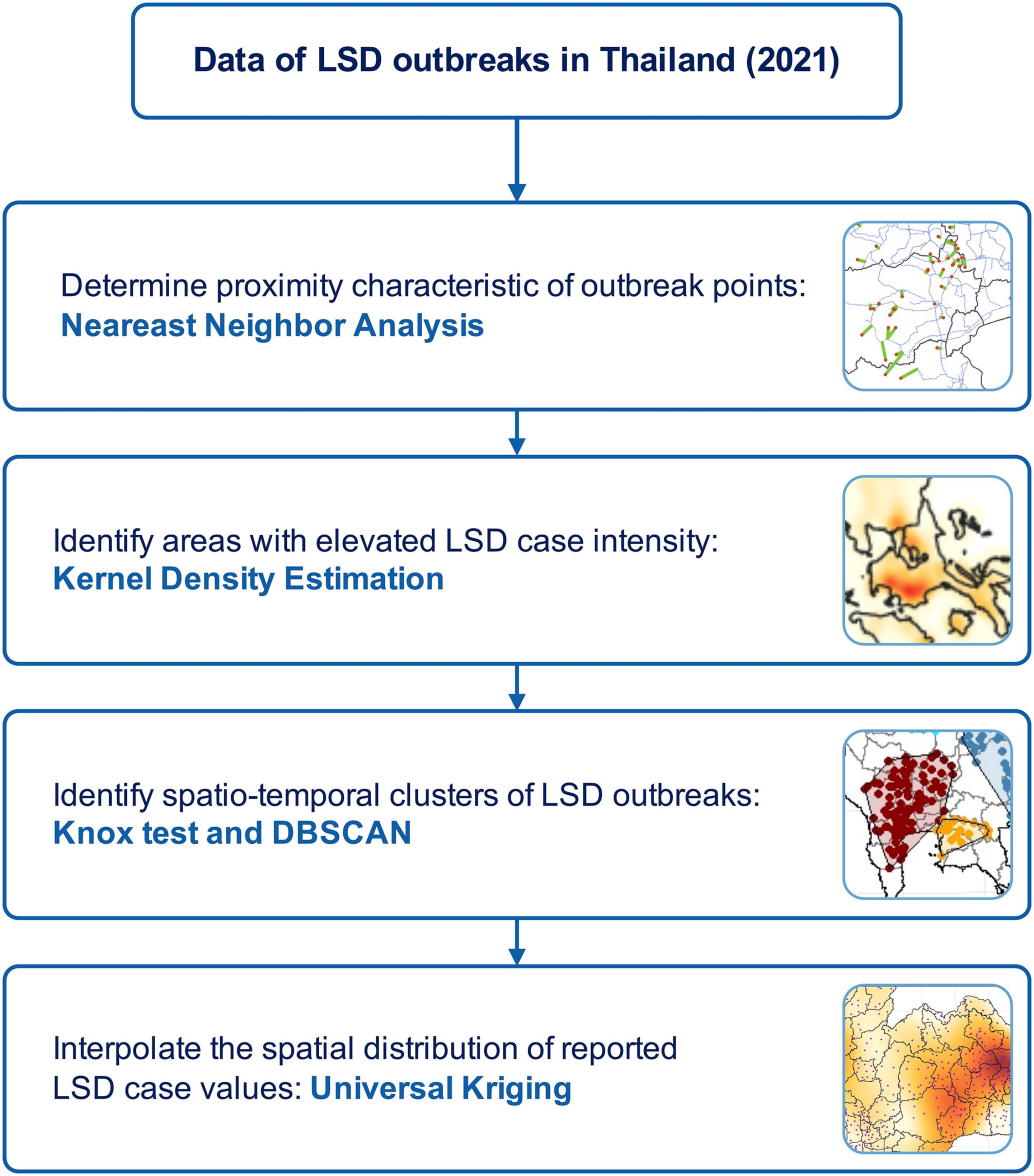
Overview of data sources, spatial methods, and their respective purposes used in the analysis of lumpy skin disease (LSD) outbreaks.

An overview of the data sources, spatial analysis methods, and corresponding purposes used in the investigation of lumpy skin disease outbreaks is presented in Figure 1.

## 3 Results

### 3.1 Spatial and temporal distribution of LSD outbreak reports

**Figure 2:**
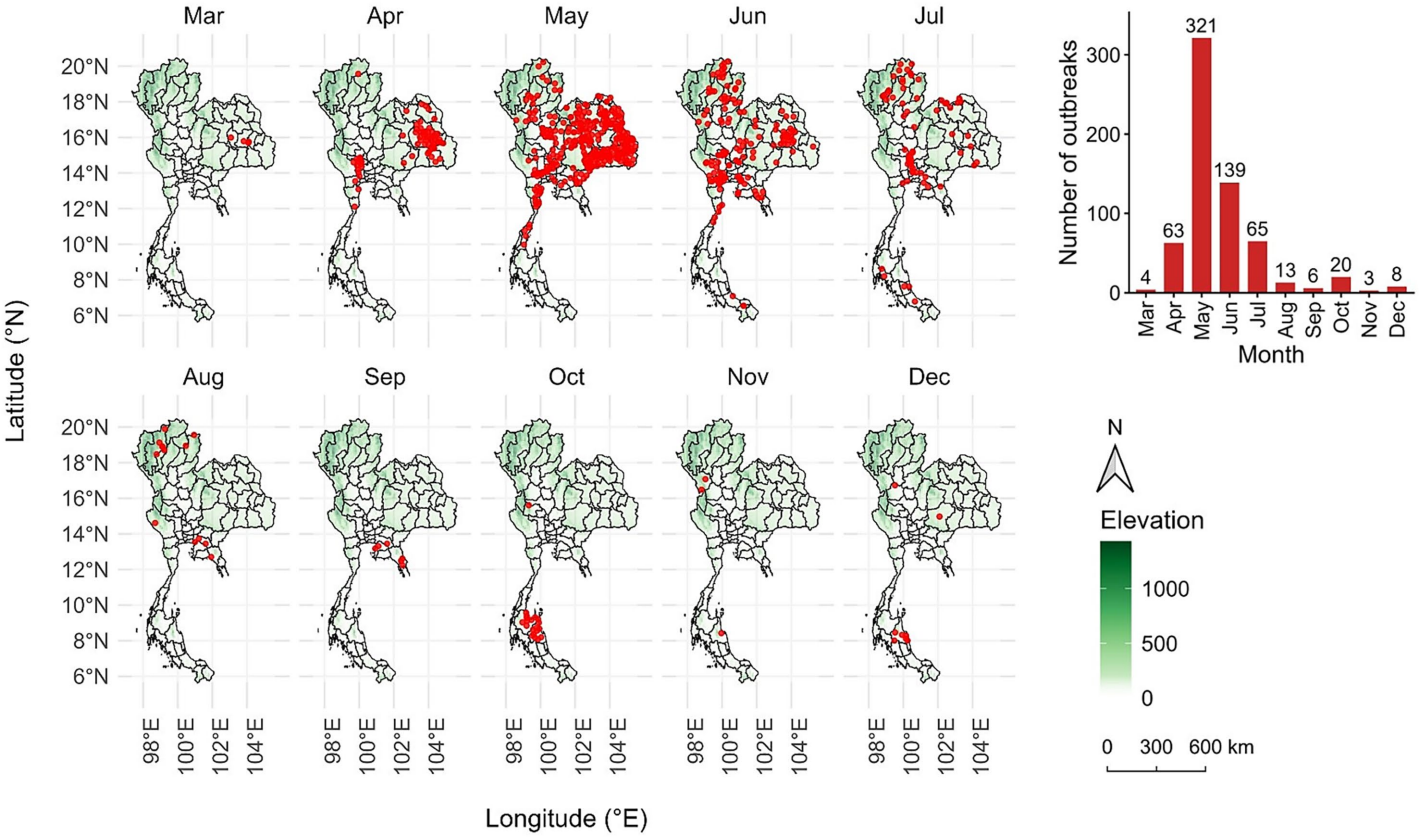
Geographical distribution of lumpy skin disease outbreaks in Thailand from March to December 2021. Red dots indicate outbreak locations based on reported latitude and longitude coordinates, whereas the underlying elevation gradient is represented by surface colors.

The spatial and temporal distribution of LSD outbreaks revealed a progression in outbreak occurrence throughout 2021 (Figure 2). The earliest outbreak reports were observed in March (*n* = 4) and were primarily concentrated in the northeastern region. During April, outbreak reports increased substantially and remained concentrated in northeastern Thailand, with additional outbreaks appearing in several provinces of the northern and central regions. The epidemic reached its highest intensity in May, when outbreaks became widespread across much of the northeastern region and expanded into central Thailand. A high level of outbreak activity persisted in June (*n* = 139), with cases continuing to occur across the northeastern and central regions and additional outbreak locations reported in the northern and southern regions. Although outbreak reports remained evident in July, the number and geographic extent of affected locations were notably reduced compared with the peak months. Following July, outbreak occurrence declined substantially. During August and September, outbreaks were largely sporadic and occurred as isolated events rather than major outbreak concentrations. Sporadic outbreaks continued to be reported during October, November, and December, primarily in localized areas of the northern and southern regions. Overall, the results demonstrate a rapid increase in outbreak occurrence from March to May, widespread geographic distribution during the peak period of May and June, and a progressive decline in both outbreak frequency and spatial extent during the latter half of the year.

### 3.2 Spatial distribution of LSD cases

**Figure 3:**
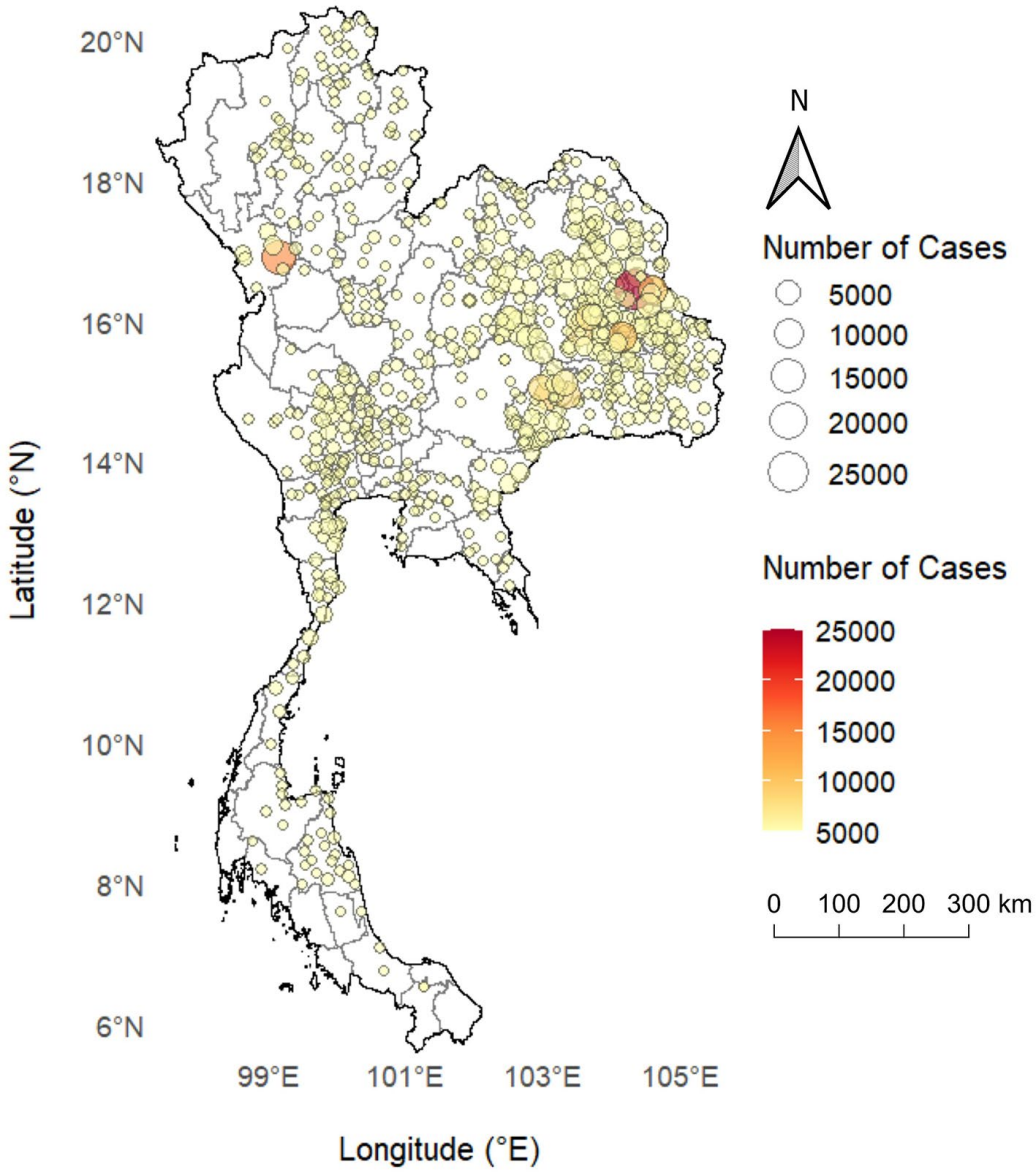
Spatial distribution of lumpy skin disease cases across Thailand, where point size represents the number of cases and color denotes the case count gradient.

The map illustrates the geographical distribution of LSD outbreaks across Thailand, with points representing the number of reported cases in different regions (Figure 3). Larger points indicate areas with higher case counts, while a color gradient from yellow (lower case numbers) to dark red (higher case numbers) visually differentiates case density across regions. The central and northern regions of Thailand exhibited the highest concentrations of LSD cases, as indicated by larger points with intense red coloration. In contrast, the southern and remote areas displayed lower case densities, marked by smaller points with yellow hues.

**Figure 4:**
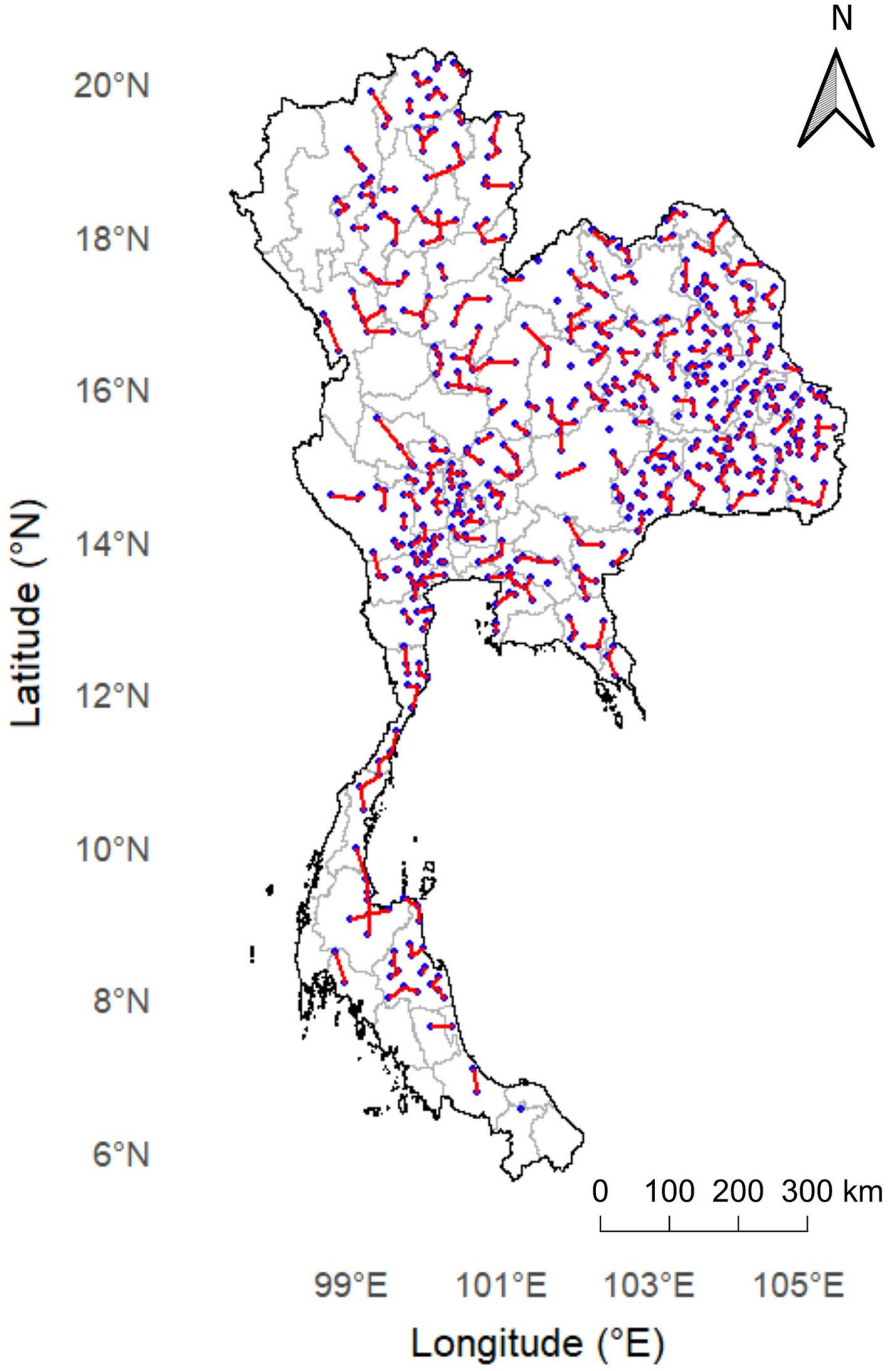
The closest distances between each outbreak location and its nearest neighboring outbreak location. Each outbreak location is represented by a blue dot, whereas the red lines indicate the shortest distances between neighboring outbreaks.

The average nearest-neighbor distance between an LSD outbreak location and its nearest neighboring outbreak was 13.75 km (median = 13.15 km, range = 0.1 km–53.77 km). Outbreak locations with short distances between neighboring sites were predominantly observed in the northeastern and west-central regions of Thailand (Figure 4).

### 3.3 Kernel density estimation of LSD outbreaks

**Figure 5:**
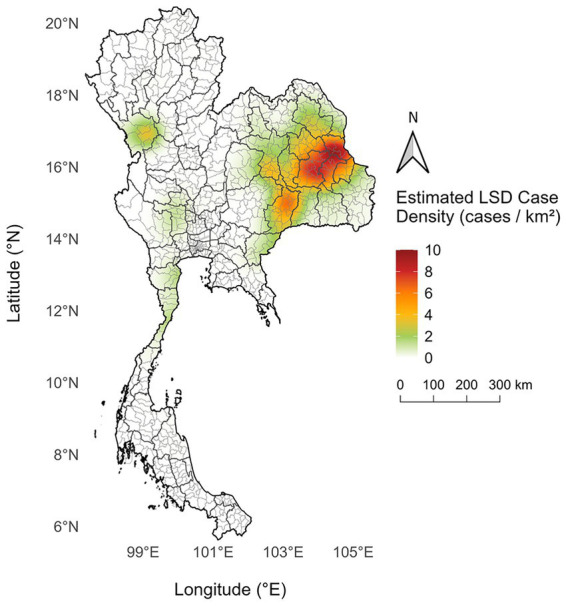
Weighted kernel density estimation (KDE) surface of reported LSD cases in Thailand during 2021. The KDE surface was generated using outbreak locations weighted by the corresponding number of reported cases. Higher values indicate geographic areas with greater concentrations of reported LSD cases, whereas lower values indicate areas with relatively sparse case occurrence. The map highlights localized concentrations of reported cases and provides a continuous representation of case density across the study area.

The kernel density estimation analysis revealed a heterogeneous spatial distribution of LSD outbreaks across Thailand (Figure 5). The highest outbreak intensity was observed in the northeastern region, where a prominent hotspot was identified in the eastern part of the region and surrounded by a broad area of moderate to high kernel density values. A second major hotspot was detected in the central region, characterized by elevated outbreak density extending across several adjacent provinces. Additional areas of moderate outbreak concentration were observed in parts of the central-northeastern region. Smaller localized hotspots were also evident in the upper northern and northwestern regions, while a limited area of elevated density was observed in the southern region. In contrast, much of the western and southern parts of the country exhibited relatively low kernel density values. The gradual transition from high- to low-density areas across the KDE surface indicates that outbreak occurrence was concentrated within several focal regions rather than being uniformly distributed across the country. Overall, the KDE analysis identified the northeastern and central regions as the principal areas of outbreak concentration, with additional localized hotspots occurring in the northern and southern regions.

### 3.4 Space–time clusters of LSD outbreaks

**Figure 6:**
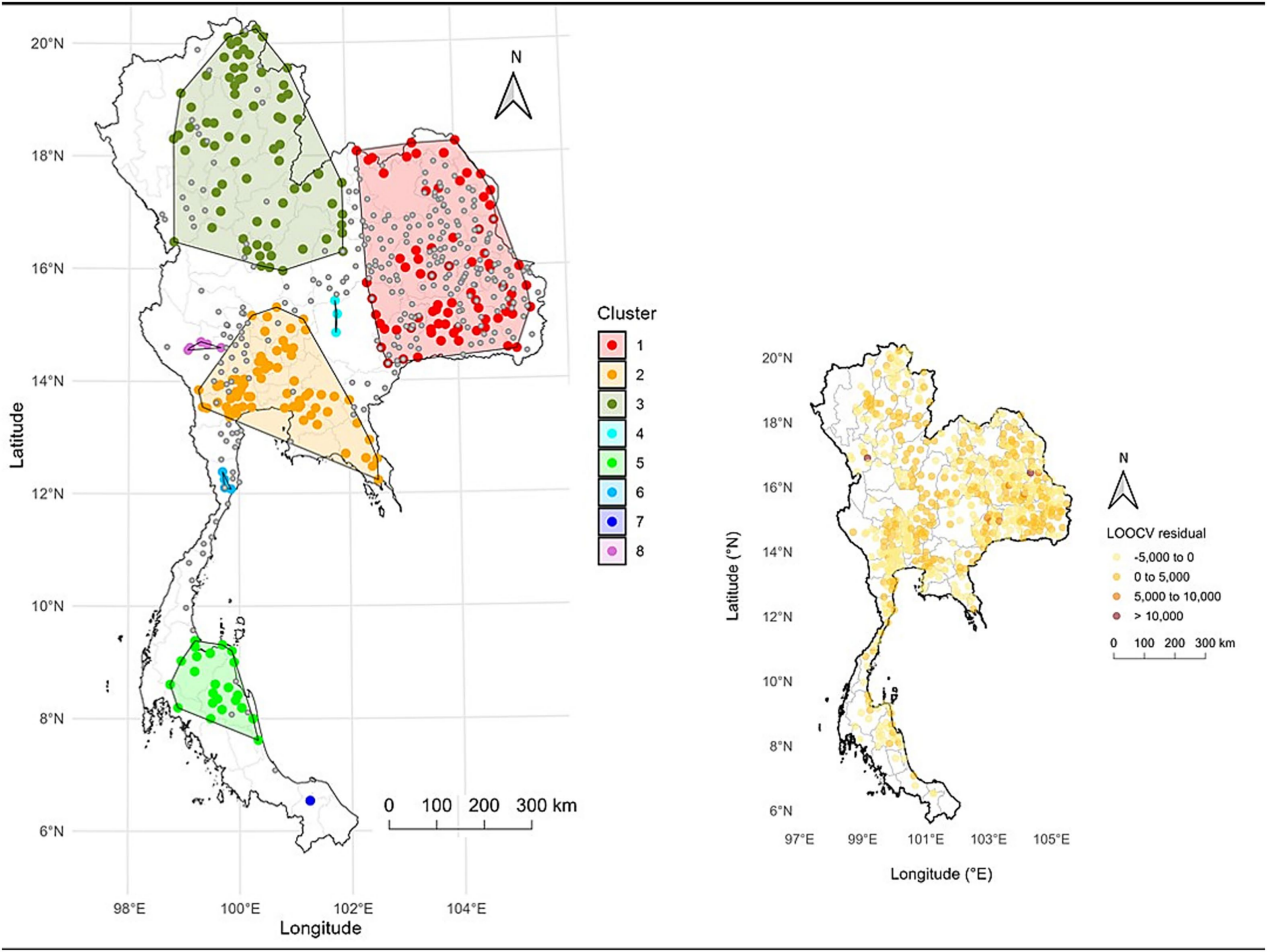
Spatio-temporal clusters of lumpy skin disease (LSD) outbreaks identified using the DBSCAN algorithm based on standardized space-time distances. Colored points and polygons represent outbreak locations assigned to the same cluster, while gray points represent outbreak locations not assigned to a cluster (Left). Spatial distribution of leave-one-out cross-validation (LOOCV) residuals from the universal kriging model used to interpolate reported LSD case values (Right).

Overall, the results demonstrate substantial heterogeneity in the size, duration, and outbreak burden of the identified spatio-temporal clusters. DBSCAN identified eight spatio-temporal clusters of LSD outbreaks across Thailand (Table 1, Figure 6). Results showed that Cluster 2 was the largest cluster and was concentrated in the northeastern region, encompassing outbreak locations distributed across multiple neighboring provinces. Cluster 1 was located in the central region and extended into adjacent western and eastern areas, forming another large cluster. Cluster 3 was situated in the northern region and represented an additional major concentration of outbreaks spanning several provinces. In contrast, Clusters 4, 6, 7, and 8 were considerably smaller and more localized. Cluster 4 was identified in the lower northern region and consisted of only a few outbreak locations. Clusters 6 and 7 occurred in the southern region, with Cluster 7 located in the far south. Cluster 8 represented a small localized cluster in the western region. Cluster 5 formed a distinct cluster in the southern region and was geographically separated from the major clusters identified in the northern, northeastern, and central parts of the country (Table 1, Supplementary Table S1). The geographic separation of these southern clusters from the principal outbreak concentrations suggests that outbreak locations were organized into several spatially distinct groups rather than a single nationwide cluster.

**Table 1:** Epidemiological and spatial characteristics of the spatio-temporal clusters of lumpy skin disease (LSD) outbreaks identified by DBSCAN in Thailand.

Cluster ID	Number of outbreak locations	Total reported cases	Nearest-neighbor distance (km)	Total population	Earliest first-outbreak report date	Latest first-outbreak report date	Cluster duration (days)
1	86	1,127	18.1	70,988	3/25/2021	7/1/2021	98
2	137	1801	15.7	91,095	4/16/2021	8/17/2021	123
3	51	674	19.8	20,107	4/30/2021	8/1/2021	93
4	3	4	18.9	85	5/17/2021	5/19/2021	2
5	23	221	20.4	946	7/5/2021	12/24/2021	172
6	4	39	14	71,661	4/22/2021	6/16/2021	55
7	4	38	17	119	6/11/2021	7/13/2021	32
8	6	106	15.2	52,206	4/16/2021	6/5/2021	50

The clusters varied considerably in their size and temporal extent. Cluster durations ranged from 2 to 172 days. Most major clusters emerged during March and April 2021, corresponding to the period of rapid epidemic expansion. The mean nearest-neighbor distance within clusters ranged from 14.0 to 20.4 km. Cluster 6 exhibited the highest spatial compactness, whereas Cluster 5 showed the most dispersed spatial configuration. The three largest clusters displayed similar nearest-neighbor distances, indicating comparable levels of spatial aggregation.

The results from the sensitivity analysis demonstrated that the identified cluster structure was sensitive to the temporal threshold selected (Supplementary Table S2). Reducing the temporal threshold to 15 days produced a larger number of smaller clusters and increased the number of unclustered outbreak locations. In contrast, temporal thresholds of 30 and 45 days resulted in fewer and larger clusters, with fewer outbreak locations classified as noise. These findings indicate that shorter temporal thresholds partition outbreak locations into more localized space–time clusters, whereas longer thresholds merge temporally proximate outbreaks into broader cluster structures. Although the number and size of clusters varied across thresholds, the major outbreak concentrations remained evident under all scenarios, suggesting that the overall spatial pattern was relatively robust to changes in the temporal threshold.

### 3.5 Universal kriging interpolation

#### 3.5.1 Semivariogram and leave-one-out cross-validation

The Gaussian variogram model provided an adequate representation of the spatial structure of the LSD case data (Table 2). Leave-one-out cross-validation indicated minimal prediction bias, as reflected by the low ME. The RMS and ASE suggested reasonable predictive performance, while the RMSS indicated a moderate tendency to underestimate prediction uncertainty. Furthermore, the positive correlation between observed and predicted values demonstrated that the model was able to capture the broad spatial pattern of LSD case distribution. Collectively, these validation results support the suitability of the kriging model for characterizing the spatial variation in LSD cases, although some local variability remained unexplained.

**Table 2:** Variogram parameters and leave-one-out cross-validation (LOOCV) results for the universal kriging model of reported LSD case values in Thailand.

Model	Gaussian
Nugget	1,500,000
Partial sill	3,000,000
Total sill	4,500,000
Range (km)	160
Practical range (km)	277.1
Mean Error (ME)	−2.03
Root Mean Square Error (RMS)	1569.19
Average Standardized Error (ASE)	1268.98
Root Mean Square Average Standardized Error (RMSS)	1.25
Correlation	0.381

#### 3.5.2 Evaluation of kriging assumptions

Moran’s *I* tests performed on the LOOCV residuals revealed no significant residual spatial autocorrelation for any neighborhood size evaluated (*k* = 4–10) (Table 3). Moran’s *I* values ranged from −0.035 to −0.001, indicating near-random spatial patterns in the residuals. All tests were non-significant (*p* = 0.482–0.915), suggesting that the spatial dependence present in the observed data was adequately accounted for by the kriging model. Consequently, no substantial residual spatial structure remained after interpolation.

**Table 3:** Moran’s *I* statistics for assessing residual spatial autocorrelation in leave-one-out cross-validation (LOOCV) residuals from the universal kriging model using Moran’*I* test.

*k*	Moran’s *I*	*p*-value
4	−0.0346	0.915
5	−0.0098	0.648
6	−0.0060	0.589
8	−0.0008	0.482
10	−0.0216	0.905

Visual examination of the LOOCV residual distribution (Figure 6) further supported these findings. Most residuals were small in magnitude and distributed throughout the study area without forming obvious geographic concentrations. Although a limited number of locations exhibited relatively large positive residuals, these residuals were spatially isolated and did not form distinct clusters. Together, the residual map and Moran’s *I* analyses indicate that the kriging model adequately captured the major spatial structure of LSD case distribution and that the remaining prediction errors were largely random in space.

#### 3.5.3 Interpolated case-value surface

**Figure 7:**
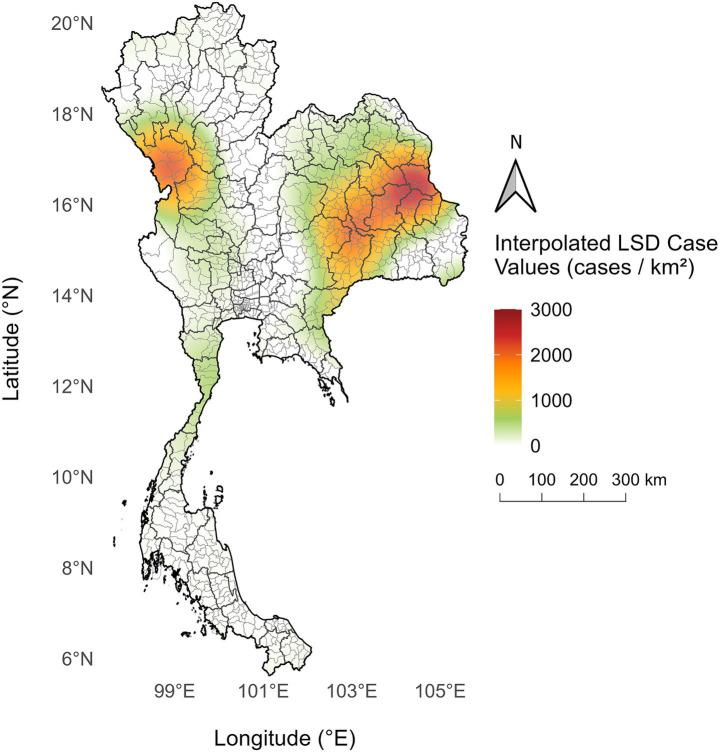
Universal kriging interpolation of reported LSD case values in Thailand during 2021. The interpolated surface was generated using reported LSD case values while accounting for cattle population as a covariate. Higher values represent areas with elevated interpolated case intensity, whereas lower values indicate areas with lower interpolated values. The map illustrates broad spatial variation in the distribution of reported LSD cases and provides a continuous representation of interpolated case values across the study area.

The interpolated surface revealed marked spatial heterogeneity in the distribution of predicted LSD case values across Thailand (Figure 7). The highest predicted values were concentrated in the northeastern region, where a large contiguous area of elevated values was observed, particularly in the eastern portion of the region. Additional areas of moderate predicted values were identified in the central region, including localized areas extending toward the lower northeastern region. Smaller areas of elevated values were also observed in the northwestern and southern regions, although these were considerably less extensive than those identified in the northeastern region. In contrast, much of the upper northern, western, and southern parts of the country exhibited low predicted values. The interpolated surface further revealed gradual spatial transitions between areas of high and low values rather than abrupt boundaries, illustrating the continuous spatial variation in the distribution of LSD cases. Overall, the kriging analysis highlighted the northeastern region as the principal area with elevated predicted case values while also identifying several smaller localized areas of moderate values that were less apparent from the observed outbreak locations alone.

## 4 Discussion

This study is the first to combine multiple spatial analytical approaches, including proximity analysis, Knox test and DBSCAN-based cluster detection, kernel density mapping, and kriging-based interpolation of the spatial distribution of LSD case values, within a unified national-scale framework to investigate LSD outbreaks in Thailand.

The movement of infected animals significantly contributes to the long-distance transmission of LSDV, whereas arthropod vectors facilitate rapid transmission over shorter distances (2, 63, 64). The relatively short average distance between outbreak locations observed in this study indicated that the outbreak sites were in close proximity to one another. Such a short average closest distance likely reflects the localized spread of LSD, wherein new outbreaks are more likely to occur near previously affected areas. This finding aligns with previous research conducted in Thailand, which demonstrated that cattle farms are often situated in close proximity, resulting in spatial clustering of outbreaks in specific regions (41). However, the observed spatial pattern may be influenced by factors such as the abundance of LSD vectors, environmental conditions, and shared risk factors, including livestock management practices and local animal movements (65, 66). Furthermore, the nationwide spread of LSD outbreaks can be attributed to the fact that this was the first recorded incidence of the disease in the country, coupled with the absence of prior immunity among cattle (19). Additionally, the unavailability of LSD vaccines during the initial months of the outbreak significantly hindered early disease control efforts (16, 67). Consequently, despite the implementation of various control measures, including restrictions on animal movements, closures of cattle markets, and application of insecticides, the disease has spread rapidly throughout the country (19). In this study, the proximity analysis between outbreak sites emphasized the need for timely and targeted control measures, as the close spatial arrangement of farms, particularly within identified outbreak clusters, can facilitate the rapid transmission of LSD (54, 68). This spatial pattern emphasizes the importance of prompt interventions to contain localized disease spread, which may also be applicable to other regions exhibiting similar geographic and environmental conditions, particularly where LSD is newly introduced.

The spatial distribution pattern derived from the KDE indicates that the northeastern region, which reported Thailand’s first LSD outbreak and has a high cattle density, subsequently experienced extensive disease spread across multiple provinces (18, 67). This widespread transmission led to the formation of densely clustered outbreak cases, reflecting how the introduction of LSDV into a naïve cattle population facilitates rapid local transmission and extensive disease dissemination. High-density areas were also observed in parts of the central region, consistent with livestock production systems characterized by large farm clusters and frequent animal movements (19). The lower density of LSD outbreaks in southern Thailand likely reflects a lower incidence of disease. The southern region, being geographically distant, likely benefited from earlier awareness and timely implementation of control measures, such as movement restrictions and vaccination, which helped limit transmission (54). These spatial patterns illustrate how disease dynamics are influenced by cattle population distribution, farming practices, and control measures, providing valuable insights into regions where LSDV is most likely to circulate and persist. However, spatial distribution alone captures only one dimension of disease epidemiology; a broader understanding is obtained by incorporating space–time analysis using the Knox method and DBSCAN algorithm.

Extending the analysis to a spatio-temporal perspective, the clustering results from the Knox method and DBSCAN algorithm reveal patterns that align with established epidemiological evidence and reflect the geographic progression observed during the actual outbreak response. The finding that DBSCAN identified prominent clusters in the northeastern, northern, and central regions is consistent with both previous studies and actual outbreak progression. Earlier investigations have documented that LSD first emerged in the northeastern region, subsequently spread to the north, and then expanded into central Thailand and neighboring provinces before eventually affecting southern areas (19). The previous national SaTScan analysis detected between 1 and 31 clusters depending on the model structure and parameter settings employed (18). In comparison, the DBSCAN analysis identified eight distinct outbreak clusters based on outbreak density and spatio-temporal proximity. Although several DBSCAN clusters occurred in geographic regions similar to those identified by SaTScan, including the northeastern, northern, and central regions of Thailand, the DBSCAN approach delineated density-connected outbreak groups with irregular spatial configurations and distinguished clustered outbreaks from isolated outbreak events. This distinction may assist veterinary authorities in differentiating core outbreak areas requiring intensive intervention from sporadic outbreak locations. Furthermore, the KDE analysis identified broader zones of outbreak concentration than those delineated by discrete clusters. While SaTScan identifies statistically significant clusters within predefined scanning windows, KDE generates a continuous outbreak intensity surface that captures gradients of disease occurrence extending beyond cluster boundaries (69). Consequently, areas adjacent to statistically significant clusters but exhibiting elevated outbreak intensity may also be identified as priorities for intervention. This information may be particularly useful when defining vaccination zones, vector-control areas, and active surveillance boundaries, as disease control measures are often implemented across contiguous geographic areas rather than strictly within the boundaries of statistically significant clusters. The kriging analysis further complemented these findings by generating a continuous risk surface and identifying areas with elevated predicted risk that did not always correspond exactly to the principal outbreak clusters detected by DBSCAN or previously reported by SaTScan. Such areas may warrant enhanced surveillance and preparedness activities despite having a lower observed outbreak density. Technically, the sensitivity analysis indicated that a 15-day temporal threshold generated a large number of small clusters, reflecting stricter temporal linkage among outbreak locations. While such a threshold may be useful for identifying short-term outbreak signals, it may fragment epidemiologically related outbreaks when retrospective outbreak data are analyzed over an extended period. In contrast, a 30-day threshold produced a more parsimonious cluster structure while preserving the principal outbreak concentrations observed during the epidemic. Therefore, the 30-day threshold was considered appropriate for characterizing broad spatio-temporal outbreak patterns and supporting retrospective epidemiological interpretation.

The interpolated surface revealed substantial spatial heterogeneity in the spatial distribution of reported LSD case values across Thailand, with the highest interpolated values concentrated in the northeastern region and smaller areas of elevated values identified in the central, northwestern, and southern regions. This pattern is consistent with the observed outbreak distribution and may reflect the combined influence of cattle population density and environmental conditions that favor vector abundance and persistence (43, 70). The elevated interpolated case values observed in the northeastern region are consistent with previous studies that identified this region as one of the principal areas affected during the 2021 LSD epidemic in Thailand.

Unlike point-based outbreak maps, the interpolated surface provided a continuous representation of the spatial distribution of reported LSD case values, highlighting gradual transitions between areas with relatively high and low interpolated values rather than discrete outbreak locations alone. Notably, the interpolation identified broader zones of elevated case intensity surrounding reported outbreak locations, suggesting that outbreak activity was not uniformly distributed but concentrated within specific geographic areas. Such information may complement cluster analyses and kernel density estimation by providing an additional perspective on the spatial distribution of cases.

Although the present study focused on the spatial epidemiology of LSD outbreaks, molecular evidence provides important context for interpreting the observed patterns. The first LSD outbreaks reported in Thailand in 2021 were caused by a recombinant LSDV strain genetically related to recombinant viruses previously identified in China and Vietnam. Whole-genome analyses demonstrated that the Thai strain possessed a mosaic genome comprising a vaccine-derived backbone interspersed with field-strain genomic fragments, suggesting the introduction and rapid dissemination of a recombinant lineage during the initial epidemic wave (19). Following the nationwide spread of the disease, mass vaccination using live attenuated LSD vaccines was initiated in June 2021 and was associated with a subsequent decline in reported cases (71). Subsequent molecular surveillance conducted during 2021–2022 confirmed that recombinant LSDV strains continued to circulate in Thailand, while genomic mutations were detected in isolates collected from different regions, including central Thailand, indicating ongoing viral evolution after vaccine deployment (72, 73). However, molecular data were not available for the outbreaks included in the present analysis; therefore, differentiation between field strains, vaccine-related strains, and recombinant variants could not be performed. Consequently, the identified spatial clusters should be interpreted as epidemiological patterns rather than evidence of transmission associated with specific viral lineages. Future studies integrating genomic surveillance and phylogeographic analyses with spatial epidemiology would strengthen source attribution and improve understanding of LSDV dissemination pathways.

## 5 Implications

The spatial analyses conducted in this study provide information that can support the planning and implementation of LSD prevention and control programs in Thailand. The identification of outbreak concentrations, spatio-temporal clusters, and areas with elevated interpolated case values can assist livestock authorities in prioritizing surveillance activities, targeting field investigations, and allocating resources to locations where outbreak occurrence has historically been concentrated. Such information may be particularly valuable when surveillance resources are limited and cannot be distributed uniformly across all regions. The findings can also support the strategic allocation of disease-control resources. Areas identified as major outbreak concentrations may be prioritized for vaccination campaigns, vector-control activities, movement-control measures, and outbreak investigations. Furthermore, the identification of geographically connected outbreak groups through the Knox and DBSCAN analyses may assist authorities in defining operational control zones and improving coordination of control activities across neighboring administrative areas. The broader outbreak-concentration zones identified by kernel density estimation may also provide useful guidance for establishing surveillance and vaccination boundaries that extend beyond individual outbreak locations.

In addition, the interpolated surface provides a continuous representation of the spatial distribution of reported LSD case values, enabling authorities to visualize broad geographic patterns and identify areas where reported cases were concentrated during the epidemic. This information may assist in preparedness planning, contingency planning, and the prioritization of future monitoring activities. Together, these analytical outputs can support evidence-based decision-making and contribute to more efficient deployment of personnel, vaccines, surveillance efforts, and financial resources during future LSD outbreaks.

## 6 Limitations

This study has several limitations. First, the analysis was based on secondary outbreak data obtained from WOAH reports, similar to previous studies (74–76); therefore, reporting bias and underreporting cannot be completely excluded. In addition, the outbreak coordinates represented village-level locations rather than exact farm coordinates. The results should therefore be interpreted at the village and regional scales rather than the individual farm level. Second, the analysis included only reported outbreak locations and did not incorporate areas without reported outbreaks, farm-level denominator data, or surveillance intensity. Consequently, areas without reported outbreaks should not be interpreted as disease-free. Similarly, the kriging output represents an interpolation of reported LSD case values rather than a direct measure of disease risk. Moreover, the KDE analysis identified concentrations of reported LSD cases and that the kriging analysis generated an interpolated surface of reported LSD case values. Neither analysis should be interpreted as a direct measure of disease risk. In addition, reported case numbers were not normalized by farm density or other farm-level denominator data, although cattle population was incorporated as a covariate in the kriging analysis, Finally, the study focused on characterizing spatial and spatio-temporal outbreak patterns through proximity analysis, clustering, density mapping, and interpolation. Variables potentially associated with outbreak occurrence, such as animal movements, vaccination coverage, vector abundance, climatic conditions, and other environmental factors, were not included. Future studies incorporating these data, together with advanced approaches such as ecological niche modeling, Bayesian spatial models, or other epidemiological risk-modeling frameworks, may provide additional insights into disease suitability, transmission dynamics, and epidemiological risk. Such approaches would complement the present study, which was designed to characterize outbreak distribution patterns and interpolate the spatial distribution of reported LSD case values rather than estimate disease risk.

## 7 Conclusion

This study integrated the nearest neighbor analysis, kernel density estimation, Knox test, and universal kriging to comprehensively assess the spatial distribution and clustering patterns of LSD outbreaks in Thailand. The results revealed areas with high case concentrations, close spatial proximity between outbreak locations, significant space–time clusters, and regions with elevated outbreak risk, including areas without reported cases. These findings provide useful evidence for enhancing surveillance strategies, guiding resource allocation, and strengthening disease control measures. Practically, the results highlight the need for prioritizing vaccination efforts in identified high-risk clusters, strengthening vector control in areas conducive to disease transmission, and enforcing movement restrictions in identified hotspots. Incorporating spatial analysis into routine epidemiological investigations can enhance preparedness and response capacity, thereby improving the overall effectiveness of outbreak prevention and control efforts against this transboundary disease.

## Data Availability

The original outbreak records analyzed in this study are publicly available through the WOAH-WAHIS database. The processed dataset generated and analyzed during this study has been deposited and is available at DOI: 10.13140/RG.2.2.14457.84328. Readers are encouraged to obtain the original outbreak records directly from the WOAH-WAHIS platform to ensure access to the primary data source. The complete R scripts used for data processing, spatial analyses, model fitting, validation procedures, and figure production are provided as Supplementary material.
