# Supplementary material for: Spatial epidemiology of lumpy skin disease outbreaks in Thailand: proximity patterns, case concentrations, spatio-temporal clusters, and interpolated case distribution
Source: Front Vet Sci. 2026 Jul 23;13:1867178. doi: 10.3389/fvets.2026.1867178 (PMC13441768; doi:10.3389/fvets.2026.1867178)
Supplement: Supplementary file 1 [file Data_Sheet_1.PDF]

R codes for KDE analysis

```
library(ggplot2)
library(sf)
library(spatstat.geom)
library(spatstat.explore)
library(terra)
library(dplyr)
library(RColorBrewer)

data_kde <- "P:/01 Work/Vee_Rmapping/Data/LSD_Asia_Thailand only_2021.csv"
data <- read.csv(data_kde)
coordinates <- data.frame(
  Longitude = data$Longitude,
  Latitude  = data$Latitude,
  cases     = data$cases_3
) %>%
  filter(!is.na(Longitude), !is.na(Latitude))
points_sf <- st_as_sf(
  coordinates,
  coords = c("Longitude", "Latitude"),
  crs = 4326,
  remove = FALSE
)

thailand <- st_read(
  "P:/01 Work/Shapefile/Country boundaries - shp/shp file THAILAND -
origin/Province/TH_Province.shp",
  quiet = TRUE
) %>%
  st_transform(4326) %>%
  st_make_valid() %>%
  st_collection_extract("POLYGON") %>%
  st_zm(drop = TRUE) %>%
  filter(!st_is_empty(geometry))

amphoe <- st_read(
  "P:/01 Work/Shapefile/Country boundaries - shp/shp file THAILAND -
origin/Amphoe_PROV/Amphoe_PROV.shp",
  quiet = TRUE
) %>%
  st_transform(4326) %>%
  st_make_valid() %>%
  st_collection_extract("POLYGON") %>%
  st_zm(drop = TRUE) %>%
  filter(!st_is_empty(geometry))

thailand_border <- thailand %>% st_union()

target_crs <- 32647
thailand_m <- st_transform(thailand, target_crs)
```

```r
amphoe_m   <- st_transform(amphoe, target_crs)
border_m   <- st_transform(thailand_border, target_crs)
points_m   <- st_transform(points_sf, target_crs)

coords_m <- st_coordinates(points_m)
win <- as.owin(border_m)
pp <- ppp(
  x = coords_m[, 1],
  y = coords_m[, 2],
  window = win,
  marks = coordinates$cases
)
summary(pp)

kde_event <- density.ppp(
  pp,
  sigma = 30000,
  eps   = 3000,
  edge  = TRUE
)
kde_event$v <- kde_event$v * 1e6
kde_case <- density.ppp(
  pp,
  sigma  = 30000,
  eps    = 3000,
  weights = marks(pp),
  edge   = TRUE
)
kde_case$v <- kde_case$v * 1e6
kde_use <- kde_case
legend_name <- "Cases / km²"

kde_df <- as.data.frame(kde_use)
colnames(kde_df) <- c("x", "y", "density")
kde_rast <- rast(
  kde_df,
  type = "xyz",
  crs = st_crs(target_crs)$wkt
)
kde_rast_mask <- mask(kde_rast, vect(border_m))
kde_plot_df <- as.data.frame(kde_rast_mask, xy = TRUE, na.rm = TRUE)
colnames(kde_plot_df) <- c("x", "y", "density")

density_map <- ggplot() +
  geom_sf(data = thailand_m, fill = "gray90", color = "black", linewidth = 0.4) +
  geom_raster(
    data = kde_plot_df,
    aes(x = x, y = y, fill = density),
    interpolate = TRUE
  ) +
  geom_sf(data = thailand_m, fill = NA, color = "black", linewidth = 0.4) +
```

```r
  scale_fill_distiller(
    palette = "YlOrRd",
    direction = 1,
    name = legend_name
  ) +
  coord_sf(crs = st_crs(target_crs)) +
  theme_minimal() +
  labs(
    title = "Kernel Density of LSD Cases in Thailand",
    x = "Easting (m)",
    y = "Northing (m)"
  )
print(density_map)

combined_map <- ggplot() +
  geom_sf(data = thailand_m, fill = "gray90", color = "black", linewidth = 0.4) +
  geom_raster(
    data = kde_plot_df,
    aes(x = x, y = y, fill = density),
    interpolate = TRUE
  ) +
  geom_sf(data = thailand_m, fill = NA, color = "black", linewidth = 0.4) +
  geom_sf(
    data = points_m,
    aes(size = cases),
    shape = 21,
    fill = "white",
    color = "grey15",
    alpha = 0.6
  ) +
  scale_fill_distiller(
    palette = "YlOrRd",
    direction = 1,
    name = legend_name
  ) +
  scale_size_continuous(
    name = "Number of cases",
    range = c(2, 8)
  ) +
  coord_sf(crs = st_crs(target_crs)) +
  theme_minimal() +
  labs(
    title = "Kernel Density and LSD Cases in Thailand",
    x = "Easting (m)",
    y = "Northing (m)"
  )
print(combined_map)

kde_rast_wgs84 <- project(
  kde_rast_mask,
  "EPSG:4326",
```

```
  method = "bilinear"
)
kde_df <- as.data.frame(
  kde_rast_wgs84,
  xy = TRUE,
  na.rm = TRUE
)
colnames(kde_df) <- c("x", "y", "z")
summary(kde_df$z)

kde_df2 <- kde_df %>%
  filter(
    is.finite(x),
    is.finite(y),
    is.finite(z)
  )

create_density_map <- function(kde_df,
                               boundaries,
                               country_border,
                               na_color = "transparent",
                               alpha_fill = 1,
                               boundary_color = "gray30",
                               boundary_size = 0.3,
                               value_limits = c(0, 10),
                               breaks = c(0, 2, 4, 6, 8, 10),
                               lat_breaks = c(6, 8, 10, 12, 14, 16, 18, 20),
                               lon_breaks = c(97, 99, 101, 103, 105),
                               district_boundaries = NULL,
                               manual_colors = c("white", "darkolivegreen3", "darkgoldenrod1",
                                                 "#FF7F00", "#CD2626", "firebrick4")) {

  bbox <- st_bbox(boundaries)

  lon_breaks <- lon_breaks[lon_breaks >= bbox["xmin"] & lon_breaks <= bbox["xmax"]]
  lat_breaks <- lat_breaks[lat_breaks >= bbox["ymin"] & lat_breaks <= bbox["ymax"]]

  p <- ggplot() +
    geom_tile(
      data = kde_df,
      aes(x = x, y = y, fill = z),
      alpha = alpha_fill
    )

  if (!is.null(district_boundaries)) {
    p <- p +
      geom_sf(
        data = district_boundaries,
        fill = NA,
        color = "grey50",
        linewidth = 0.1,
```

```
        alpha = 0.5
      )
  }

  p <- p +
    geom_sf(
      data = boundaries,
      fill = NA,
      color = boundary_color,
      linewidth = boundary_size
    ) +
    geom_sf(
      data = country_border,
      color = "black",
      linewidth = 0.4,
      fill = NA
    ) +
    scale_fill_gradientn(
      colours = manual_colors,
      name = "Estimated LSD case\nintensity (cases / km²)\n",
      limits = value_limits,
      breaks = breaks,
      oob = scales::squish,
      na.value = na_color
    ) +
    scale_x_continuous(
      breaks = lon_breaks,
      labels = paste0(lon_breaks, "°E"),
      limits = c(bbox["xmin"], bbox["xmax"]),
      expand = c(0, 0)
    ) +
    scale_y_continuous(
      breaks = lat_breaks,
      labels = paste0(lat_breaks, "°N"),
      limits = c(bbox["ymin"], bbox["ymax"]),
      expand = c(0, 0)
    ) +
    labs(
      title = " ",
      x = "\nLongitude (°E)",
      y = "Latitude (°N)\n"
    ) +
    theme_minimal() +
    theme(
      plot.title = element_text(size = 16, face = "bold", hjust = 0.5),
      axis.title = element_text(size = 14),
      axis.text = element_text(size = 12),
      legend.title = element_text(size = 14),
      legend.text = element_text(size = 12),
      panel.grid = element_blank()
    )
```

```
  return(p)
}

p <- create_density_map(
  kde_df = kde_df2,
  boundaries = thailand,
  district_boundaries = amphoe,
  country_border = thailand_border,
  manual_colors = c("white", "darkolivegreen3", "darkgoldenrod1",
                    "#FF7F00", "#CD2626", "firebrick4"),
  na_color = "gray80",
  value_limits = c(0, 10),
  breaks = c(0, 2, 4, 6, 8, 10)
)

p


ggsave(
  filename = "Figure3_KDE_Thailand_with_Districts.jpg",
  plot = p,
  width = 7, height = 7, units = "in", dpi = 600, bg = "white"
)
```

R-code for Kriging and DBSCAN analysis

```
library(sf)
library(dplyr)
library(geosphere)
library(ggplot2)
library(surveillance)
library(dbscan)
library(viridis)

K <- read.csv("P:/01 Work/Vee_Rmapping/Data/LSD_Asia_Thailand only_2021.csv"
data <- read.csv(data_kde)
)

thailand_sf <- st_read("P:/pCloudShare/01_Work/Shapefile/Country boundaries - shp/shp file
THAILAND/Province/TH_Province.shp")
thailand_border <- st_union(thailand_sf)  # Union to combine all provinces

points_sf <- K %>%
  st_as_sf(coords = c("x", "y"), crs = 4326)

points_sf$time <- as.Date(points_sf$time, format = "%m/%d/%Y")
points_sf$time <- as.numeric(difftime(points_sf$time, min(points_sf$time), units = "days"))

points_df <- data.frame(
  x = st_coordinates(points_sf)[, 1],  # Longitude
  y = st_coordinates(points_sf)[, 2],  # Latitude
  time = points_sf$time,             # Time in days
  observed = points_sf$observed       # Number of cases as the observed variable
)

time_dist_matrix <- dist(points_df$time)

coords <- st_coordinates(points_sf)
n <- nrow(coords)
distance_matrix_km <- matrix(0, nrow = n, ncol = n)

# Calculate spatial distances using Haversine formula
for (i in 1:(n - 1)) {
  for (j in (i + 1):n) {
    distance_matrix_km[i, j] <- distHaversine(coords[i,], coords[j,]) / 1000  # Convert to km
    distance_matrix_km[j, i] <- distance_matrix_km[i, j]  # Symmetric matrix
  }
}

# Combine temporal and spatial distances into a single distance measure
# Using 50 km (spatial) and 30 days (temporal) as explicit thresholds
time_threshold <- 30  # Temporal threshold in days
distance_threshold <- 50  # Spatial threshold in km

# Normalize distances by the thresholds
```

```r
scaled_time_dist_matrix <- time_dist_matrix / time_threshold
scaled_distance_matrix_km <- distance_matrix_km / distance_threshold

# Extract the upper triangular part of both matrices
upper_triangle_indices <- upper.tri(scaled_distance_matrix_km)
scaled_distance_matrix_km_upper <- scaled_distance_matrix_km[upper_triangle_indices]
scaled_time_dist_matrix_upper <- scaled_time_dist_matrix[upper_triangle_indices]

# Now check if the lengths match
if (length(scaled_distance_matrix_km_upper) != length(scaled_time_dist_matrix_upper)) {
  stop("The lengths of the upper triangular parts of the distance matrices do not match!")
}

# Combine the scaled distances for both spatial and temporal components
combined_distance_vector <- sqrt(scaled_distance_matrix_km_upper^2 +
scaled_time_dist_matrix_upper^2)

# Initialize counter for pairs within the thresholds
valid_pairs_count <- 0

# Count pairs within the time and distance thresholds, handling NA values
for (i in 1:length(scaled_distance_matrix_km_upper)) {
  # Check for NA values before comparing
  if (!is.na(scaled_time_dist_matrix_upper[i]) && !is.na(scaled_distance_matrix_km_upper[i])) {
    if (scaled_time_dist_matrix_upper[i] <= 1 && scaled_distance_matrix_km_upper[i] <= 1) {  #
Check if both thresholds are met
      valid_pairs_count <- valid_pairs_count + 1  # Increment valid pairs counter
    }
  }
}

cat("Number of valid pairs within 30 days and 50 km thresholds:", valid_pairs_count, "\n")

# Number of points
n <- length(points_df$time)

# Reshape the combined distance vector into a square distance matrix
combined_distance_matrix <- matrix(0, nrow = n, ncol = n)
combined_distance_matrix[upper.tri(combined_distance_matrix)] <-
combined_distance_vector
combined_distance_matrix[lower.tri(combined_distance_matrix)] <-
t(combined_distance_matrix)[lower.tri(combined_distance_matrix)]

# Check and replace NA values with Inf
combined_distance_matrix[is.na(combined_distance_matrix)] <- Inf

# Check and replace Inf values with a large number (to avoid DBSCAN issues)
combined_distance_matrix[is.infinite(combined_distance_matrix)] <- 1e10

# Perform DBSCAN with adjusted eps and minPts to detect more clusters
# Dynamically adjust eps to allow more clusters
```

```
eps_value <- max(median(combined_distance_vector, na.rm = TRUE) * 2, 0.1)  # Increase eps
by a factor of 2 (but not below 0.1)

# Further reduce minPts to allow even smaller clusters
minPts_value <- 2  # Reduce minPts to 2 to allow even more clusters (instead of 3)

# Perform DBSCAN with the adjusted eps and minPts
dbscan_result <- dbscan(combined_distance_matrix, eps = eps_value, minPts = minPts_value)

# Summary of DBSCAN results
# Table of points per cluster
cluster_table <- table(points_df$cluster)
# Number of noise points (cluster 0)
num_noise <- cluster_table["0"]
# Total number of clusters (excluding noise)
num_clusters <- length(cluster_table) - ifelse("0" %in% names(cluster_table), 1, 0)
# Print results
cat("Number of clusters (excluding noise):", num_clusters, "\n")
cat("Number of noise points (cluster 0):", num_noise, "\n\n")
cat("Points per cluster:\n")
print(cluster_table)

# ---------------------------------------------------------------------------
# Define a Sensitivity Function
library(dbscan)
library(dplyr)

run_dbscan_sensitivity <- function(points_df, coords, base_time_vector, time_threshold,
spatial_threshold = 50, minPts = 2, save_path = NULL, plot_suffix = NULL) {

 # Temporal distance
 time_dist_matrix <- dist(base_time_vector)

 # Spatial distance matrix
 n <- nrow(coords)
 distance_matrix_km <- matrix(0, nrow = n, ncol = n)
 for (i in 1:(n - 1)) {
  for (j in (i + 1):n) {
   distance_matrix_km[i, j] <- distHaversine(coords[i,], coords[j,]) / 1000
   distance_matrix_km[j, i] <- distance_matrix_km[i, j]
  }
 }

 # Scale
 scaled_time_dist <- as.matrix(time_dist_matrix) / time_threshold
 scaled_spatial_dist <- distance_matrix_km / spatial_threshold

 # Combine distances
 combined_dist <- sqrt(scaled_time_dist^2 + scaled_spatial_dist^2)
 combined_dist[is.na(combined_dist)] <- Inf
 combined_dist[is.infinite(combined_dist)] <- 1e10
```

```r

  # Determine eps dynamically
  upper_vals <- combined_dist[upper.tri(combined_dist)]
  eps_val <- max(median(upper_vals, na.rm = TRUE) * 2, 0.1)

  # Run DBSCAN
  dbscan_result <- dbscan(combined_dist, eps = eps_val, minPts = minPts)
  clusters <- as.factor(dbscan_result$cluster)

  # Summary
  cluster_table <- table(clusters)
  num_noise <- ifelse("0" %in% names(cluster_table), cluster_table["0"], 0)
  num_clusters <- length(cluster_table) - ifelse("0" %in% names(cluster_table), 1, 0)

  # Output
  cat("===== Temporal threshold:", time_threshold, "days =====\n")
  cat("Number of clusters:", num_clusters, "\n")
  cat("Number of noise points:", num_noise, "\n")
  print(cluster_table)
  cat("\n")

  # Save as CSV
  if (!is.null(save_path)) {
    out_df <- as.data.frame(cluster_table)
    colnames(out_df) <- c("Cluster", "Count")
    out_df$Temporal_Threshold <- time_threshold
    write.csv(out_df, file = paste0(save_path, "dbscan_summary_", time_threshold, "d.csv"),
row.names = FALSE)
  }

  # Return cluster labels
  return(clusters)
}
# Define thresholds
thresholds <- c(15, 30, 45)

# Save path
save_folder <- "P:/pCloudShare/01_Work/DBSCAN_Sensitivity/"

# Run for each threshold
for (t in thresholds) {
  points_df[[paste0("cluster_", t, "d")]] <- run_dbscan_sensitivity(
    points_df = points_df,
    coords = coords,
    base_time_vector = points_df$time,
    time_threshold = t,
    spatial_threshold = 50,
    minPts = 2,
    save_path = save_folder
  )
}
```

```
# Get the unique clusters (including 0 for unclustered points)
unique_clusters <- unique(points_sf$cluster)
unique_clusters

# Display outbreak records belonging to each cluster
cluster_records <- points_sf %>%
  st_drop_geometry() %>%
  select(
    cluster,
    Outbreak_id,
    Location_name,
    observed,
    susceptible_3,
    time
  ) %>%
  arrange(cluster, time)

print(cluster_records)

# Cluster characteristics
# Keep original date
points_sf$date <- as.Date(K$time, format="%m/%d/%Y")

# Create numeric time for DBSCAN
points_sf$time <- as.numeric(
  difftime(points_sf$date,
           min(points_sf$date),
           units = "days")
)
cluster_stats <- points_sf %>%
  st_drop_geometry() %>%
  group_by(cluster) %>%
  summarise(
    n_outbreaks = n(),
    total_cases = sum(observed, na.rm = TRUE),
    total_susceptible = sum(susceptible_3, na.rm = TRUE),
    start_date = min(date),
    end_date = max(date),
    duration_days = as.numeric(max(date) - min(date))
  ) %>%
  arrange(as.numeric(as.character(cluster)))

print(cluster_stats)

# Add DBSCAN cluster assignments to points_df
points_df$cluster <- as.factor(dbscan_result$cluster)

# Now add the cluster info back into the points_sf object (sf format)
points_sf <- points_sf %>%
  mutate(cluster = points_df$cluster)  # Adding cluster info to the spatial object
```

```
# Calculate convex hull for each cluster, excluding cluster 0
convex_hull_data <- points_sf %>%
  group_by(cluster) %>%
  summarise(geometry = st_union(geometry)) %>%  # Union points in each cluster
  st_make_valid() %>%                    # Ensure valid geometry before computing convex hull
  st_convex_hull() %>%                   # Calculate convex hull for valid geometries
  filter(cluster != 0)                   # Exclude cluster 0 from the convex hull data

# Get the unique clusters (including 0 for unclustered points)
unique_clusters <- unique(points_sf$cluster)

# Ensure that we have enough colors for all clusters (excluding unclustered points)
# Refined color values based on your figure
# Define colors for clusters 1–8
color_values <- c(
  "red",        # Cluster 1
  "orange",     # Cluster 2
  "olivedrab",   # Cluster 3 (olive green)
  "cyan",       # Cluster 4
  "green",   # Cluster 5 (yellow-orange)
  "deepskyblue", # Cluster 6 (sky blue)
  "blue",       # Cluster 7 (deep blue)
  "orchid"      # Cluster 8 (light magenta)
)

# Get unique cluster IDs
unique_clusters <- sort(unique(points_sf$cluster))
cluster_levels <- unique_clusters[unique_clusters != 0]  # Exclude cluster 0 (unclustered)

# Extend color palette if needed
if (length(cluster_levels) > length(color_values)) {
  color_values <- c(color_values, rep("gray", length(cluster_levels) - length(color_values)))
}

# Plot the spatial distribution
ggplot() +
  # Add map layers
  geom_sf(data = thailand_sf, fill = "white", color = "gray", linewidth = 0.05) +
  geom_sf(data = thailand_border, fill = NA, color = "black", size = 0.5) +

  # Clustered outbreak points
  geom_sf(data = points_sf %>% filter(cluster != 0),
          aes(color = as.factor(cluster)), size = 2) +

  # Convex hulls
  geom_sf(data = convex_hull_data %>% filter(cluster != 0),
          aes(fill = as.factor(cluster)), color = "black", alpha = 0.2) +

  # Unclustered points (cluster 0) in gray50
  geom_sf(data = points_sf %>% filter(cluster == 0),
          color = "gray50", fill = "gray75", shape = 21, size = 1, show.legend = FALSE) +
```

```
  # Labels and theme
  labs(
    x = "Longitude", y = "Latitude",
    color = "Cluster", fill = "Cluster"
  ) +
  theme_minimal() +
  theme(legend.position = "right") +

  # Manual scales for clusters
  scale_color_manual(values = setNames(color_values, cluster_levels)) +
  scale_fill_manual(values = setNames(color_values, cluster_levels))

# --------- Add Cluster number on the map
# Plot with cluster labels
ggplot() +
  # Add Thailand boundary
  geom_sf(data = thailand_sf, fill = "white", color = "gray50", linewidth = 0.05) +
  geom_sf(data = thailand_border, fill = NA, color = "black", size = 0.5) +

  # Plot clustered points
  geom_sf(data = points_sf %>% filter(cluster != 0),
          aes(color = as.factor(cluster)), size = 2) +

  # Plot convex hulls
  geom_sf(data = convex_hull_data %>% filter(cluster != 0),
          aes(fill = as.factor(cluster)), color = "black", alpha = 0.2) +

  # Plot unclustered points (cluster == 0) as gray dots without legend
  geom_sf(data = points_sf %>% filter(cluster == 0),
          color = "gray40", fill = "gray40", shape = 21, size = 2, show.legend = FALSE) +

  # Add cluster number labels at centroids
  geom_text(data = cluster_centroids,
            aes(x = st_coordinates(centroid)[,1],
                y = st_coordinates(centroid)[,2],
                label = cluster),
            color = "red", size = 8, fontface = "bold") +

  # Titles and axis labels
  labs(title = "Spatial Clusters of Outbreak Locations (DBSCAN Clustering)",
       subtitle = "Clusters based on DBSCAN from Knox Test Pairs",
       x = "Longitude", y = "Latitude",
       color = "Cluster", fill = "Cluster") +

  # Theme and legend
  theme_minimal() +
  theme(legend.position = "right") +

  # Manual color scales
  scale_color_manual(values = setNames(color_values, cluster_levels)) +
```

```
scale_fill_manual(values = setNames(color_values, cluster_levels))
```
