# Supplementary material for: Spatial epidemiology of lumpy skin disease outbreaks in Thailand: proximity patterns, case concentrations, spatio-temporal clusters, and interpolated case distribution
Source: Front Vet Sci. 2026 Jul 23;13:1867178. doi: 10.3389/fvets.2026.1867178 (PMC13441768; doi:10.3389/fvets.2026.1867178)
Supplement: Supplementary file 2 [file Table_1.PDF]

Supplementary-1

Supplementary Table S1. Provinces and regions represented within the spatio-temporal clusters of lumpy skin disease (LSD) outbreaks identified by DBSCAN in Thailand during 2021.

Cluster Number	Province	Region
1	Amnat Charoen, Bueng Kan, Buri Ram, Chaiyaphum, Kalasin, Maha Sarakham, Mukdahan, Nakhon Phanom, Nakhon Ratchasima, Nong Khai, Roi Et, Sakon Nakhon, Si Sa Ket, Surin, Ubon Ratchathani, Yasothon, Udon Thani.	North East
2	Ang Thong, Bangkok Metropolis, Buri Ram, Chachoengsao, Chaiyaphum, Chanthaburi, Chon Buri, Kanchanaburi, Loei, Lop Buri, Nakhon Nayok, Nakhon Pathom, Nakhon Sawan, Nakhon Si Thammarat, Pathum Thani, Phetchabun, Phichit, Phitsanulok, Phra Nakhon Si Ayutthaya, Ratchaburi, Sa Kaeo, Samut Prakan, Samut Sakhon, Samut Songkhram, Saraburi, Sing Buri.	Central, East-Central
3	Chiang Mai, Chiang Rai, Lampang, Lamphun, Nan, Phayao, Phrae, Uttaradit.	Northern
4	Loei, Phetchabun	North East
5	Krabi, Nakhon Si Thammarat, Phatthalung, Songkhla, Surat Thani.	Southern
6	Prachuap Khiri Khan	
7	Songkhla, Yala	Southern
8	Nakhon Pathom	Western

Supplementary Table S2. Distribution of outbreak locations among DBSCAN clusters using alternative temporal thresholds (15 and 45 days).

Temporal threshold 15 days

Cluster	Count
0	22
1	408
2	10
3	3
4	2
5	3
6	48
7	10
8	19
9	2
10	10
11	2
12	5
13	2

14	2
15	3
16	3
17	4
18	6
19	2
20	2
21	2

Temporal threshold 50 days

0	10
1	449
2	12
3	3
4	52
5	26
6	10
7	2
8	6
